# Partial nucleotide sequence analysis of the VP6 gene from rotavirus species A, D, and F identified in diarrheic broiler chickens

**DOI:** 10.1007/s42770-026-01932-w

**Published:** 2026-04-22

**Authors:** Jéssica Cristhine Gallego, Elis Lorenzetti, Elisabete Takiuchi, Alice Fernandes Alfieri, Amauri Alcindo Alfieri

**Affiliations:** 1 Department of Preventive Veterinary Medicine, Laboratory of Animal Virology, Universidade Estadual de Londrina, Celso Garcia Cid Road, PR-445 Km 380, P.O. Box 10011, Londrina, PR 86057-970 Brazil; 2 Department of Preventive Veterinary Medicine, Multi-User Animal Health Laboratory, Molecular Biology Unit, Universidade Estadual de Londrina, Celso Garcia Cid Road, PR-445 Km 380, P.O. Box 10011, Londrina, PR 86057-970 Brazil; 3 Post Graduate Program in Animal Health and Production, Universidade Pitágoras Unopar Anhanguera, Arapongas, PR Brazil; 4 Department of Veterinary Sciences, Universidade Federal do Paraná, Palotina, PR Brazil

**Keywords:** Poultry, Diarrhea, Malabsorption syndrome, Avian rotavirus, Genotype I

## Abstract

Rotavirus (RV) causes viral enteric diseases in several livestock species. It is a non-enveloped, icosahedral virus with 11 segments of double-stranded RNA genome encoding six structural proteins (VP). VP6, the most abundant protein, is located in the middle layer of the RV capsid and is commonly used for detection and classification of RV species. Avian RVs are classified into four species: RVA, RVD, RVF, and RVG, with RVA being the most frequently reported in avian hosts, though it remains little studied. We aimed to identify RV species and possible co-infections, characterize the VP6 gene in fecal samples previously confirmed as RV-positive by silver-stained polyacrylamide gel electrophoresis, and to evaluate their phylogenetic and diversity relationships. The sampling included 55 RV-positive fecal samples from 1–2-week-old broiler chickens exhibiting diarrhea and clinical signs of malabsorption syndrome. Nucleic acids were extracted and subjected to reverse transcription-polymerase chain reaction assays targeting the VP6 gene of RVA, RVD, RVF, and RVG. Amplified products were sequenced, and 33 nucleotide (nt) sequences were submitted for phylogenetic analysis. RVA, RVD, and RVF species were identified in 10 (18.2%), 17 (30.9%), and 28 (50.9%) of the fecal samples, respectively. Only one RV species was identified in each fecal sample. RVG VP6 gene was not amplified in any of the fecal samples. Phylogenetic analysis revealed that the 10 RVA strains clustered within genotype I11. RVD and RVF strains showed 87.7–90.1% and 87.9–98.4% nt identity, respectively. Molecular characterization of circulating RV strains in avian species aids in understanding the epidemiology of these infections.

## Introduction

Rotavirus (RV) infection is the primary viral etiology of diarrhea in young animals worldwide, including mammals and avian species [1]. In addition to children, mammal infections have clinical and epidemiological significance in several species of livestock animals, particularly calves [2] and piglets [3].

Avian RV is a common cause of enteric infection in avian species, leading to diarrhea and dehydration [4]. In broiler chickens, RV infection is associated with various clinical signs, collectively termed malabsorption syndrome or runting-stunting syndrome [5]. This syndrome reduces weight gain, batch non-uniformity, and high culling rates [6]. In Brazil, the occurrence of avian RV has been previously evaluated in both symptomatic [7–10] and asymptomatic [11–16] broilers and layers.

Based on the viral protein VP6, present in the middle layer of the capsid, 11 RV species (*Rotavirus alphagastroenteritidis* (RVA), *Rotavirus betagastroenteritidis* (RVB), *Rotavirus tritogastroenteritidis* (RVC), *Rotavirus deltagastroenteritidis* (RVD), *Rotavirus phigastroenteritidis* (RVF), *Rotavirus gammagastroenteritidis* (RVG), *Rotavirus aspergastroenteritidis* (RVH), *Rotavirus iotagastroenteritidis* (RVI), *Rotavirus jotagastroenteritidis* (RVJ), *Rotavirus kappagastroenteritidis* (RVK), *Rotavirus lambdagastroenteritidis* (RVL)) have been described to date [17]. Four RV species (RVA, RVD, RVF, and RVG) have been identified in avian species [5]. RVA infection occurs in mammals and avian species, whereas the RV species D, F, and G occur exclusively in avian hosts [1]. The limited availability of VP6 (genotype I) nucleotide (nt) sequences for avian RVD, RVF, and RVG in public databases hampers genotype assignment and restricts our understanding of the genetic diversity and evolutionary dynamics of these viruses [18]. However, for avian RVA field strains, the genotype I can be determined through phylogenetic analysis of the VP6 gene. Unlike RV strains identified in mammals, molecular characterization of avian RV field strains remains limited [18].

The first VP6 genotype identified in an avian RVA strain was the I4 genotype in studies conducted in the United Kingdom [19], Japan [20], Germany [21], and Nigeria [22]. Recently, RVA genotype I4 was described in domestic pigeons from Poland [23]. RVA genotype I11 was described in Ireland [24], Germany [6, 21, 24, 25], and Nigeria [22]. The I21 genotype was identified from wild Japanese common gull fecal samples [26]. A previous study conducted in Brazil with fecal samples from commercial chicken reported I4 and I11 genotypes in RVA field strains [15].

This study aimed to identify circulating RV species using reverse transcription–polymerase chain reaction (RT-PCR) and to investigate RV co-infections. Partial VP6 gene sequences from avian RV field strains, previously identified as RV-positive by silver-stained polyacrylamide gel electrophoresis (ss-PAGE), were further characterized to assess genetic diversity and phylogenetic relationships.

## Materials and methods

### Ethics approval

The experimental procedure follows the ethical principles compiled by the Brazilian College of Animal Experimentation (COBEA) and was approved by the Animals Use Ethics Committee (CEUA/protocol number 15/2014) of the Universidade Federal do Paraná.

### Biological samples

Fifty-five individual intestinal content samples from small-sized broiler chicks (runts) of the *COBB 500* commercial breed, aged 1–2 weeks, showing diarrhea and clinical signs of malabsorption syndrome were previously identified as RV-positive using ss-PAGE [10]. All fecal samples were obtained from 10 poultry flocks in Paraná State, southern Brazil, and were stored at -80 °C until processing.

### Nucleic acid extraction

Suspensions of 10% (w/v) of the fecal samples in Tris/Calcium buffer (10 mM Tris–HCl; 1.5 mM CaCl_2_; pH 7.3) were centrifuged at 5,000 × *g* for 10 min. Aliquots of 500 µL of supernatant were treated with sodium dodecyl sulfate (1% final concentration) and incubated for 30 min at 56 °C. A combination of the phenol/chloroform/isoamyl alcohol (25:24:1) and silica/guanidine isothiocyanate methods [27] was used for nucleic acid extraction. Nucleic acid was eluted in 50 µL of ultrapure sterile water treated with diethylpyrocarbonate (DEPC) (Invitrogen Life Technologies, CA, USA) and stored at -80 °C until used in RT-PCR assays. DEPC water was used as a negative control and positive samples for each avian RV species were used as positive controls during the nucleic acid extraction and RT-PCR assay procedures.

### RT-PCR assay

To confirm the presence of avian RV species and to detect possible RV co-infections, extracted nucleic acids were subjected to four parallel singleplex RT-PCR assays using species-specific primers (Table 1). These assays partially amplified VP6 gene fragments of 787 bp, 741 bp, 875 bp, and 1,034 bp corresponding to avian RVA [21], RVD [28], RVF, and RVG [14], respectively. RT-PCR amplicons were analyzed through electrophoresis on a 2% agarose gel in TBE buffer pH 8.4 (89mM Tris, 89mM boric acid, and 2mM EDTA) containing ethidium bromide (0.5 µg/mL) and visualized under UV light.

**Table 1:** Specific reverse transcription-polymerase chain reaction primers used for amplifying the VP6 gene and sequencing of avian rotavirus field strain sequence, including information on expected amplicon sizes, genome position based on prototype strains, and references

Avian rotavirus species	Amplicon size	Nucleotide position *	Reference
RVA	787 bp	317 to 1,104	[21]
RVD	741 bp	24 to 765	[28]
RVF	875 bp	53 to 928	[14]
RVG	1,034 bp	11 to 1,045	[14]

* According to the reference strains: RVA Ch-06V0661 (GenBank accession number EU486969); RVD 06V0047 (GenBank accession number JN034679); RVF 03V0568 (GenBank accession number NC_021635); and RVG 03V0567 (GenBank accession number NC_021588)

### Sequencing and phylogenetic analysis

RT-PCR amplicons corresponding to RVA (*n* = 10), RVD (*n* = 12), and RVF (*n* = 11) were purified using the PureLink^®^ Quick Gel Extraction and PCR Purification Combo kit (Invitrogen Life Technologies) and quantified in a Qubit™ fluorometer using Quant-iT™ dsDNA BR Assay Kit (Invitrogen Life Technologies). Sequencing reactions were performed in both directions with the same forward and reverse primers used in RT-PCR assays in an ABI3500 Genetic Analyzer sequencer with BigDye^®^ Terminator v3.1 Cycle Sequencing Kit (Applied Biosystems, CA, USA). The nt sequence quality analysis was performed using PHRED software and the consensus sequences were assembled using the CAP3 software (http://asparagin.cenargen.embrapa.br/phph/). The nt sequence similarity searches were performed with sequences deposited in GenBank using the Basic Local Alignment Search Tool (http://blast.ncbi.nlm.nih.gov/) software. Additionally, nt sequences were submitted to the Virus Pathogen Database and Analysis Resource (http://www.viprbrc.org/) automated genotyping tool to confirm the avian RVA genotypes [29]. The nt and amino acid (aa) sequences were aligned with representative strains from other RV species that were retrieved from GenBank with ClustalW [30]. Criteria for exclusion of NCBI available sequences included: unpublished journals, sequencing technologies other than Sanger dideoxy sequencing, and sequences shorter than 300 nt. Additionally, we selected representative sequences from the same study. The phylogenetic tree was obtained using the maximum composite likelihood (ML) method with Tamura-3-parameter plus Gamma distribution + invariable sites (T92 + G + I) model with 1,000 bootstrap replicates using the MEGA software package (Version 10.2.6) [31]. The nt substitution model was determined using the “Find Best Model” analysis in MEGA software. The RVA, RVD, and RVF nt and aa identity matrices were generated using BioEdit software version 7.2.5 [32].

## Results

Of the 55 individual fecal samples evaluated by RT-PCR assays, the presence of avian RVA, RVD, and RVF species was confirmed in 10 (18.2%), 17 (30.9%), and 28 (50.9%) samples, respectively. The VP6 gene of the RVG species was not detected in any of the samples analyzed. All RV-positive samples contained RNA from only one avian RV species.

Table 2 presents the percentage comparisons of nt and aa identities between the avian RV field strains described in this study and reference prototype RV strains. It also includes a comparison with other Brazilian and world strains of avian RV belonging to RVA, RVD, and RVF species. The identity matrix analysis for RVA, RVD, and RVF species correspond to 729 nt (349 to 1,077 nt) and 243 aa (117 to 359 aa); 561 nt (183 to 743 nt) and 186 aa (55 to 240 aa); and 645 nt (92 to 736 nt) and 215 aa (23 to 237 aa), respectively.

**Table 2:** Percentage of nucleotide and amino acid identities between the avian rotavirus (RV) field strains of this study, compared with the reference prototype strain, and with other Brazilian strains of avian RV belonging to RVA, RVD, and RVF species from the state of Paraná, southern Brazil

Avian rotavirus species	Percentage identity		
	Nucleotide (nt)		Amino acid (aa)
RVA	729 nt (349 to 1,077)		243 aa (117 to 359)
This study RVA I11 strains	96 to 100%		100%
RVA reference I11 strain ^**#**^	90.9 to 92.3%		96.2%
Other Brazilian RVA I11 strains	94.7 to 97.5%		97.5 to 100%
Other world RVA I11 strains	92.7 to 94.5%		99.1 to 100%
RVD	561 nt (183 to 743)		186 aa (55 to 240)
This study RVD strains	98.9 to 100%		100%
RVD reference strain ^**#**^	88.0 to 88.2%		98.9%
Other Brazilian RVD strains	87.7 to 89.3%		100%
Other world RVD strains	88.0 to 90.1%		98.9 to 100%
RVF	645 nt (92 to 736)		215 aa (23 to 237)
This study RVF strains	97.6 to 100%		99.5 to 100%
RVF reference strain ^**#**^	87.9 to 88.5%		94.8 to 95.3%
Other Brazilian RVF strains	92.8 to 98.4%		98.6 to 100%
Other world RVF strains	89.3 to 90%		97.2 to 97.6%

^**#**^ Reference strains: RVA (strain Ch-06V0661, GenBank accession number EU486969), RVD (strain 06V0047, GenBank accession number JN034679), RVF (strain 03V0568, GenBank accession number NC_021635)

The deduced aa sequence of 10 avian RVA field strains was compared with the reference prototype strain (Ch-06V0661 - GenBank accession number: EU486969). The field strains were found to have nine aa substitutions (128 Y→N; 140 D→N; 141 M→ L; 181 C→W; 183 T→N; 200 D→N; 202 H→Q; 214 V→L; 258 K→N). Comparing the deduced aa sequence of the RVA strains from this study with a Brazilian 22/08 strain (GenBank accession number: KX198692) collected in 2008, showed 94.7 to 95.1% nt identity and 97.5% aa identity, plus identified six distinct aa divergences (238 I→V; 250 D→E; 272 V→N; 284 N→K; 285 F→K; 316 N→T). With the Korean AvRV-2 strain (GenBank accession number: JQ085406) collected in 2011 presented 92.7 to 93.5% nt identity and 99.1% aa identity, and two aa divergences (188 I→L and 327 D→E) and presented 93.1 to 94.1% nt identity and 99.5% aa identity and one aa divergence (202 Q→H) with the German Ch-03V0158G3 strain (GenBank accession number: EU486966) (Table 3).

**Table 3:** Partial comparative analysis of the VP6 protein of avian rotavirus A strains corresponding to 117 to 359 amino acids (aa) and the schematic presentation of the localization of aa substitutions. The numbers above correspond to the aa position compared with reference strain Ch-06V0661 (GenBank accession number: EU486969). The sequences obtained in this study are marked in bold plus ◼ symbol

	Amino acid position number																
Avian rotavirus strains	1	1	1	1	1	1	2	2	2	2	2	2	2	2	2	3	3
	2	4	4	8	8	8	0	0	1	3	5	5	7	8	8	1	2
	8	0	1	1	3	8	0	2	4	8	0	8	2	4	5	6	7
Prototype - EU486969/RVA/Ch-wt/DEU/Ch-06V0661/2006/I11	Y	D	M	C	T	I	D	H	V	I	D	K	V	N	F	N	D
◼ **MZ964147/RVA/Ch-wt/BRA/VP6APR_103/2015/G19-I11**	N	N	L	W	N	.	N	Q	L	.	.	N	.	.	.	.	.
◼ **OR165102/RVA/Ch-wt/BRA/VP6APR_203/2015/I11**	N	N	L	W	N	.	N	Q	L	.	.	N	.	.	.	.	.
◼ **OR165103/RVA/Ch-wt/BRA/VP6APR_206/2015/I11**	N	N	L	W	N	.	N	Q	L	.	.	N	.	.	.	.	.
◼ **OR165104/RVA/Ch-wt/BRA/VP6APR_210/2015/I11**	N	N	L	W	N	.	N	Q	L	.	.	N	.	.	.	.	.
◼ **OR165105/RVA/Ch-wt/BRA/VP6APR_299/2015/I11**	N	N	L	W	N	.	N	Q	L	.	.	N	.	.	.	.	.
◼ **OR165106/RVA/Ch-wt/BRA/VP6APR_305/2015/I11**	N	N	L	W	N	.	N	Q	L	.	.	N	.	.	.	.	.
◼ **OR165107/RVA/Ch-wt/BRA/VP6APR_312/2015/I11**	N	N	L	W	N	.	N	Q	L	.	.	N	.	.	.	.	.
◼ **OR165108/RVA/Ch-wt/BRA/VP6APR_313/2015/I11**	N	N	L	W	N	.	N	Q	L	.	.	N	.	.	.	.	.
◼ **OR165109/RVA/Ch-wt/BRA/VP6APR_316/2015/I11**	N	N	L	W	N	.	N	Q	L	.	.	N	.	.	.	.	.
◼ **OR165110/RVA/Ch-wt/VP6APR_319/BRA/2015/I11**	N	N	L	W	N	.	N	Q	L	.	.	N	.	.	.	.	.
KX198699/RVA/Av-wt/BRA/198/15/2015/G19-P[31]-I11	N	N	L	W	N	.	N	Q	L	.	.	N	.	.	.	.	.
KX198690/RVA/Av-wt/BRA/1/09/2009/I11	N	N	L	W	N	.	N	Q	L	.	.	N	.	.	.	.	.
KX198692/RVA/Av-wt/BRA/22/08/2008/G19-P[31]-I11	N	N	L	W	N	.	N	Q	L	V	E	N	N	K	K	T	.
EU486968/RVA/Ch-wt/DEU/Ch-04V0027G6/2004/G19-P[30]-I11	N	N	L	W	N	.	N	Q	L	.	.	N	.	.	.	.	.
EU486966/RVA/Ch-wt/DEU/Ch-03V0158G3/2003/I11	N	N	L	W	N	.	N	.	L	.	.	N	.	.	.	.	.
FJ169858/RVA/Ch-tc/DEU/02V0002G3/2002/G19-P[30]-I11	N	N	L	W	N	.	N	Q	L	.	.	N	.	.	.	.	.
JQ085406/RVA/Ch-wt/KOR/AvRV-2/2011/G19-P[30]-I11	N	N	L	W	N	L	N	Q	L	.	.	N	.	.	.	.	E
KC593402/RVA/Ch-wt/KOR/ADL101778-AvRV/2010/G19-P[30]-I11	N	N	L	W	N	.	N	Q	L	.	.	N	.	.	.	.	.
KU372648/RVA/Guinea_Fowl-wt/NIE/NIE13_A_1145/2013/I11	N	N	L	W	N	.	N	Q	L	.	.	N	.	.	.	.	.
KU372557/RVA/Guinea_Fowl-wt/NIE/NIE13_A_1149/2013/I11	N	N	L	W	N	.	N	Q	L	.	.	N	.	.	.	.	.
KU372570/RVA/Ch-wt/NIE/NIE11_A_250/2011/G19-P[X]-I11	N	N	L	W	N	.	N	Q	L	.	.	N	.	.	.	.	.

*DEU* Germany, *BRA* Brazil, *KOR* Korea, *NIE* Nigeria, *Ch* chicken, *Av* Avian, *wt* wild-type, *tc* tissue cultured

The 12 RVD strains showed some similarity to the prototype 06V0047 reference strain (GenBank accession number: JN034679), with two aa substitutions (93 V→I; and 230 E→D). Comparing the deduced aa sequence of RVD strains from this study with the Indian strain, 2012 (GenBank accession number: JX187435) showed 90.0 to 90.1% nt identity and 98.9% aa identity with two aa divergences (149 M→T and 228 I→M) and compared with the Bangladesh strain, 2010 (GenBank accession numbers: JN034683) presented 88.2 to 88.4% nt identity and 98.9% aa identity with two aa divergences (95 V→A; 102 V→I) (Table 4).

**Table 4:** Partial comparative analysis of the VP6 protein of avian rotavirus D strains corresponding to 55 to 240 amino acids (aa) and the schematic presentation of the localization of aa substitutions. The numbers above correspond to the aa position compared with reference strain 06V0047 (GenBank accession number: JN034679). The sequences obtained in this study are marked in bold plus ⬤ symbol

	Amino acid position number					
Avian rotavirus strains			1	1	2	2
	9	9	0	4	2	3
	3	5	2	9	8	0
Prototype - JN034679/RVD/Ch-wt/DEU/06V0047/2006	V	V	V	M	I	E
Prototype - NC_014516/RVD/Ch-tc/DEU/05V0049/2005	I	.	.	.	.	D
⬤ **OR188340/RVD/Ch-wt/BRA/VP6DPR_23/2015**	I	.	.	.	.	D
⬤ **OR188341/RVD/Ch-wt/BRA/VP6DPR_271/2015**	I	.	.	.	.	D
⬤ **OR188342/RVD/Ch-wt/BRA/VP6DPR_274/2015**	I	.	.	.	.	D
⬤** OR188343/RVD/Ch-wt/BRA/VP6DPR_275/2015**	I	.	.	.	.	D
⬤ **OR188344/RVD/Ch-wt/BRA/VP6DPR_276/2015**	I	.	.	.	.	D
⬤ **MZ935731/RVD/Ch-wt/BRA/VP6DPR_277/2015**	I	.	.	.	.	D
⬤ **OR188345/RVD/Ch-wt/BRA/VP6DPR_279/2015**	I	.	.	.	.	D
⬤ **OR188346/RVD/Ch-wt/BRA/VP6DPR_280/2015**	I	.	.	.	.	D
⬤ **OR188347/RVD/Ch-wt/BRA/VP6DPR_281/2015**	I	.	.	.	.	D
⬤ **OR188348/RVD/Ch-wt/BRA/VP6DPR_283/2015**	I	.	.	.	.	D
⬤ **OR188349/RVD/Ch-wt/BRA/VP6DPR_285/2015**	I	.	.	.	.	D
⬤ **OR188350/RVD/Ch-wt/BRA/VP6DPR_289/2015**	I	.	.	.	.	D
MN648930/RVD/Parrot-wt/BRA/AVE52/2018	I	.	.	.	.	D
KC623164/RVD/Ch-wt/BRA/109/PA/2011	I	.	.	.	.	D
KC689307/RVD/Ch-wt/BRA/AVRVBR2/2010	I	.	.	.	.	D
JQ065735/RVD/Ch-wt/BRA/27/PA/2008	I	.	.	.	.	D
KU372618/RVD/Ch-wt/NIE/NIE13_A_1040/2013	I	.	.	T	.	E
JN034682/RVD/Ch-wt/NDL/10V0133/2010	I	.	.	.	.	D
JX187435/RVD/Ch-wt/IND/UKD48/2012	I	.	.	T	M	D
JN034683/RVD/Ch-wt/BGD/BS-7/2010	I	A	I	.	.	D
JN034685/RVD/Ch-wt/BGD/MJ-5/2010	I	.	I	.	.	D

*DEU* Germany, *BRA* Brazil, *NIE* Nigeria, *NDL* Netherlands, *IND* India, *BGD* Bangladesh, *Ch* chicken, *wt* wild-type, *tc* tissue cultured

However, the 11 RVF field strains and prototype RVF strain (GenBank accession number: NC_021635) had ten aa substitutions (30 I→V; 51 V→I; 60 T→A; 110 T→M; 121 T→A; 164 I→V; 171 A→P; 175 N→D; 226 G→D; 237 V→I). The VP6FPR_70 strain (GenBank accession number: OR165114) showed an additional aa substitution at position 25 (S→F). Comparing the deduced aa sequence of RVF strains from this study with the Brazilian strains, two distinct aa (51 I→V and 237 I→V) were detected with the AVRVFBR03 strain, 2009 (GenBank accession number: KF926655) with 92.8 to 93.6% nt identity and 98.6 to 99.0% aa identity. Comparing with the Italian 956_1 strain (GenBank accession number: KT073228) were detected five aa divergences (45 Q→H; 57 T→I; 204 Q→R; 230 S→T; 237 I→V) and 89.3 to 90.0% nt identity and 97.2 to 97.6% aa identity (Table 5).

**Table 5:** Partial comparative analysis of the VP6 protein of avian rotavirus F strains corresponding to 23 to 237 amino acids (aa) and the schematic presentation of the localization of aa substitutions. The numbers above correspond to the aa position compared with reference strain 03V0568 (GenBank accession number: NC_021635). The sequences obtained in this study are marked in bold plus **▼** symbol

	Amino acid position number														
Avian rotavirus strains							1	1	1	1	1	2	2	2	2
	2	3	4	5	5	6	1	2	6	7	7	0	2	3	3
	5	0	5	1	7	0	0	1	4	1	5	4	6	0	7
Prototype - NC_021635/RVF/Ch-wt/DEU/03V0568/2003	S	I	Q	V	T	T	T	T	I	A	N	Q	G	S	V
▼ **OR165111/RVF/Ch-wt/BRA/VP6FPR_03/2015**	.	V	.	I	.	A	M	A	V	P	D	.	D	.	I
▼ **OR165112/RVF/Ch-wt/BRA/VP6FPR_05/2015**	.	V	.	I	.	A	M	A	V	P	D	.	D	.	I
▼ **OR165113/RVF/Ch-wt/BRA/VP6FPR_63/2015**	.	V	.	I	.	A	M	A	V	P	D	.	D	.	I
▼ **OR165114/RVF/Ch-wt/BRA/VP6FPR_70/2015**	F	V	.	I	.	A	M	A	V	P	D	.	D	.	I
▼ **OR165115/RVF/Ch-wt/BRA/VP6FPR_168/2015**	.	V	.	I	.	A	M	A	V	P	D	.	D	.	I
▼ **OR165116/RVF/Ch-wt/BRA/VP6FPR_172/2015**	.	V	.	I	.	A	M	A	V	P	D	.	D	.	I
▼ **MZ964148/RVF/Ch-wt/BRA/VP6FPR_179/2015**	.	V	.	I	.	A	M	A	V	P	D	.	D	.	I
▼ **OR165117/RVF/Ch-wt/BRA/VP6FPR_182/2015**	.	V	.	I	.	A	M	A	V	P	D	.	D	.	I
▼ **OR165118/RVF/Ch-wt/BRA/VP6FPR_236/2015**	.	V	.	I	.	A	M	A	V	P	D	.	D	.	I
▼ **OR165119/RVF/Ch-wt/BRA/VP6FPR_239/2015**	.	V	.	I	.	A	M	A	V	P	D	.	D	.	I
▼ **OR165120/RVF/Ch-wt/BRA/VP6FPR_267/2015**	.	V	.	I	.	A	M	A	V	P	D	.	D	.	I
MN648929/RVF/Parrot-wt/BRA/AVE52/2018	.	V	.	I	.	A	M	A	V	P	D	.	D	.	I
OL688646/RVF/Ch-wt/BRA/BJ12/2018	.	V	.	I	.	A	M	A	V	P	D	.	D	.	I
MF361096/RVF/Ch-wt/BRA/ROTAFBRZ07/2013	.	V	.	I	.	A	M	A	V	P	D	.	D	.	I
KP824808/RVF/Ch-wt/BRA/81/2010	.	V	.	I	.	A	M	A	V	P	D	.	D	.	I
KF926655/RVF/Ch-wt/BRA/AVRVFBR03/2009	.	V	.	.	.	A	M	A	V	P	D	.	D	.	.
KT073228/RVF/Par-wt/ITA/956_1/2010	.	V	H	I	I	A	M	A	V	P	D	R	D	T	.

*DEU* Germany, *BRA* Brazil, *ITA* Italy, *Ch* Chicken, *Par *Partridge, *wt* wild-type

**Fig. 1:**
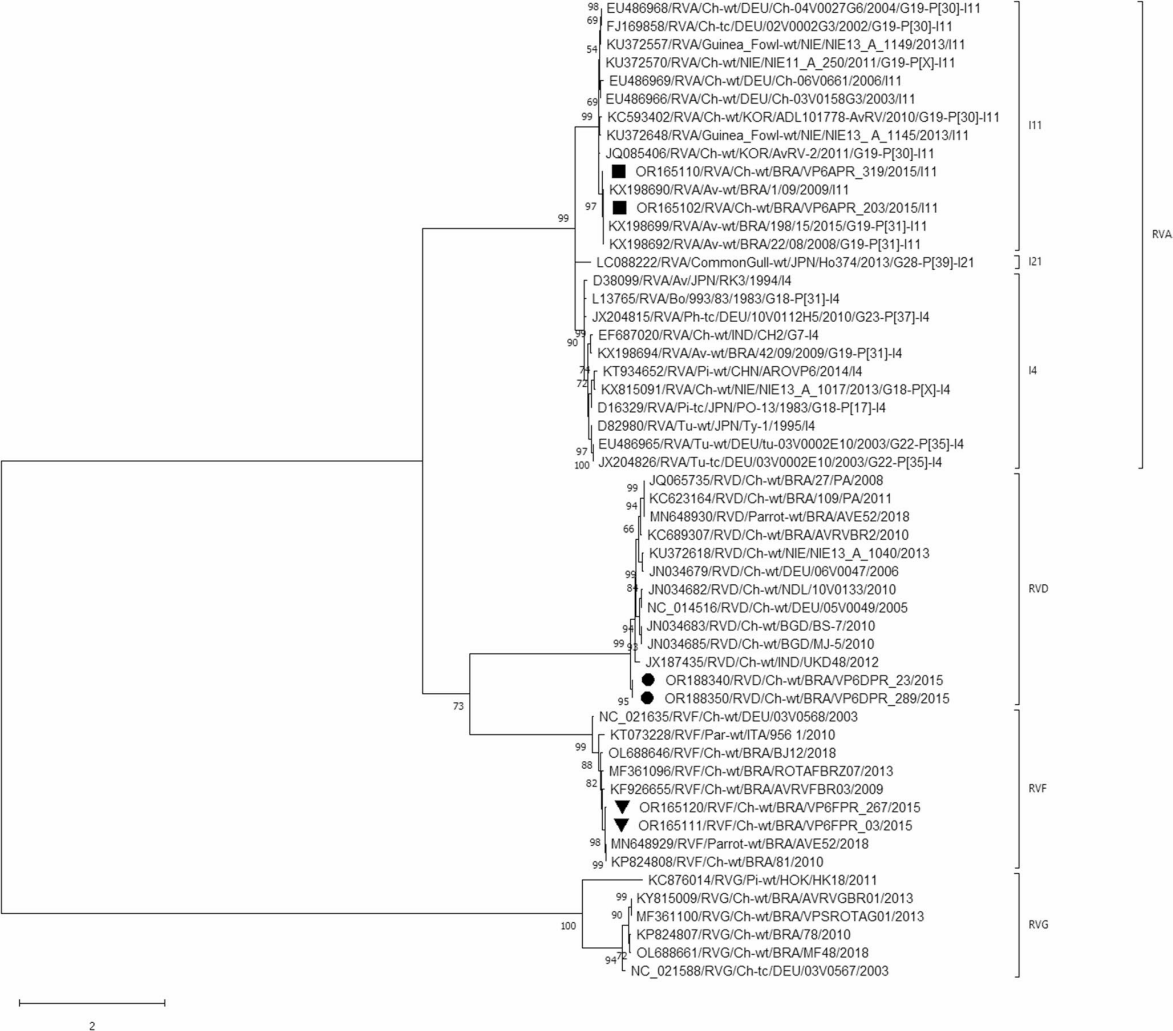
Phylogenetic tree based on the partial 386 (356 to 741) nucleotide (nt) sequences of the VP6 gene from the avian rotavirus species A (RVA), D (RVD), and F (RVF) described in this study and representative strains from avian RV species. The tree was constructed using the Maximum likelihood method with the Tamura 3-parameter model plus gamma distribution + invariable sites for nt substitution. Bootstrap values greater than 50% are shown. GenBank accession numbers of the strains are provided. VP6 nt sequences from this study are indicated with a black square, circle, and inverted triangle for the avian RVA, RVD, and RVF, respectively. Two strains represent analyzed sequences of the 10 RVA, 12 RVD, and 11 RVF wild-type strains. The VP6 gene sequences of the RVG were used as an outgroup

For the phylogenetic tree, two described field strains of each RV species were selected. The RVA strains described in this report belonged to the I11 genotype. The RVD strains clustered together to form a separate branch (subcluster) from the RVD strains already described, and the RVF strains clustered with two Brazilian RVF strains (GenBank accession numbers: KP824808 and MN648929) previously described (Fig. 1).

The partial VP6 nt sequences of the avian RVA, RVD, and RVF strains described in this study are available in GenBank under the accession numbers OR165102 to OR165110 and MZ964147, OR188340 to OR188350 and MZ935731, and OR165111 to OR165120 and MZ964148, respectively.

## Discussion

RV are recognized as significant enteric pathogens in poultry, contributing to diarrhea, growth retardation, and economic losses in commercial flocks worldwide. However, information on the classical and molecular epidemiology of RV infections in commercial and wild birds is limited [18, 22, 33, 34]. In this study, we identified and characterized avian RV species A, D, and F from diarrheic broiler chickens, providing molecular insights into the VP6 gene of circulating strains in Brazilian poultry production.

We identified three of the four RV species known to cause enteric infections in birds. All wild-type RV strains examined were obtained from commercial broiler chicken flocks showing clinical signs of malabsorption syndrome. The presence of RVA, RVD, and RVF RNA in these symptomatic chicks underscores the significance of these avian RV species in contributing to this syndrome, which is believed to have multiple causes [5, 6].

Compared to other RV species previously described in birds (RVA, RVD, and RVF), RVG is reported less frequently [14, 33]. However, cases of RVG infections in birds have been documented in several countries [35, 36], including Brazil [14, 16, 37]. RVG was not detected in any of the 55 fecal samples evaluated by RT-PCR assay in this study. In previous reports of RVG diagnosis in Brazil, samples were obtained from pools of feces and primarily collected from adult and asymptomatic birds [33, 36]. We believe that the sampling methodology may have contributed to the lack of RVG detection [10]. Moreover the potential factors can cause this, including the age of sampled birds, the presence or absence of clinical signs of diarrhea and dehydration, sample type (individual intestinal contents, individual fecal samples, or pooled fecal samples), and the diagnostic methods employed [10, 36].

Co-detection of nucleic acids from more than one avian RV species in the same biological sample using RT-PCR is common. Several reports describe the co-detection of different species of avian RV in commercial and wild birds worldwide [33, 36], including Brazil [12, 37, 38]. However, in this study, no more than one avian RV species was co-detected per intestinal content sample. Considering that the diagnostic methodology was the same (RT-PCR assay), the sample collection method may have contributed to these varying results. In some studies, biological samples were collected individually [6, 10, 38] or as pooled samples [14, 33, 36]. The age range of the evaluated birds varies widely, and analyses include symptomatic [7, 9, 10] and asymptomatic birds [9, 13–16, 38]. Our study included only fecal samples from young (first and second weeks of life) and symptomatic (diarrheic) chicks. This sampling methodology may have favored the identification of singular infections.

In this study, the partial sequence analysis of the VP6 gene of Brazilian avian RVA field strains presented a slightly low (90.9 to 92.3% nt identity) degree of genetic variability in comparison with the Ch-06V0661 prototype strain [21] and with other avian RVA sequences from Brazil (94.7 to 97.5% nt identity). Other avian RVA strains from Germany, Korea, and Nigeria that belonged to the I11 genotype ranged 92.7 to 94.5% nt identity [21, 22, 39]. Furthermore, six and nine aa divergences were present when compared with I11 genotype strains from Brazilian strain 22/08 [15] and the reference strain [21], respectively. The low identity and more aa divergences with the prototype strains can be due to the collection date. The prototype is from 2006 and the Brazilian wild strains are more recent from 2008, 2009, and 2015 years [15, 21].

Phylogenetic analysis confirmed the presence of distinct clades for each VP6 genotype of avian RVA, supported by high bootstrap values. Our RVA strains grouped inside and near other avian RV strains described in previous studies from Brazil. The VP6 partial phylogenetic analysis demonstrates that the avian RVA strains from this study clustered with other I11 genotype strains previously described from Brazil during the period 2008, 2009, and 2015 years from broiler and layer commercial farms [15] and other countries, such as Germany in 2002 to 2006 [21, 25], Korea in 2010 and 2011 [39, 40], and Nigeria in 2011 and 2013 [22]. One different host species like guinea fowl from Nigeria also grouped in I11 genotype [22].

Two genotypes (I4 and I11) of avian RVA strains have been identified in chick and turkey poults from Germany and Brazil [15, 21, 25, 41]. Two genotypes were related to Japanese strains, I21 from a common gull and I4 from a pigeon [26, 42]. The histidine residue at position 153 is involved in zinc binding, necessary for VP6 trimer structure, and conserved in all RVA isolates but not in the other RV species [43]. His153 was conserved in all analyzed sequences of avian RVA Brazilian strains.

The RVD sequences showed two and one or two aa divergences compared to the reference and worldwide RVD strains, respectively. Although the nt (87.7 to 90.1%) to identities has been slightly lower with RVD strains, the aa analysis revealed high similarity with the VP6 RVD reference with 98.9% [44], Brazilian with 100% [13, 28, 34, 45], and worldwide with 98.9 to 100% [22, 44, 46, 47]. RVD strains from this study showed nt identities comparable to temporally related strains from Brazil and other countries, such as Nigeria [22] and India [47], suggesting limited genetic divergence over time. The Brazilian RVD strains from layer chicken, 2010-year collection date showed 87.7 to 87.8% nt identity [45] and RVD strains broiler chicken, 2008- and 2011-year collection date [13, 28] showed high nt identity with 88.9 to 89.3% compared with strains described herein, respectively. With the RVD Brazilian strains from this study the higher (89.1 to 89.3%) nt identity was detected with the Parrot host collection date 2018 [34] and with broiler chicken collected in 2011 [13]. The disponible strains showed lower diversity possibly because all are temporarily close with these study samples, collected in 2015.

The Brazilian RVF strains described herein showed the lower nt and aa similarities with the German prototype strain collected in 2003 [35], and with the only worldwide strain published on GenBank, the Italian detected in a partridge game bird host collected in 2010 [36]. A similarity with strains previously described in Brazil varied between 92.8 and 98.4% and 98.6 to 100% for nt and aa, with one aa divergence (25 S→F) at VP6FPR_70 strain [12, 14, 16, 34, 37]. The nt identity from Brazilian strains don’t keep up with hosts and time patterns. The analysis showed high values 97.3 to 98.2% from 2010 collected from broiler farms [14] and 97.5 to 98.4% from 2018 year collected from wild parrot [34]. The lower nt identities were detected in Brazilian RVF strains collected in 2018 from asymptomatic broiler with 93.1 to 94.2% nt identity [16], in 2013 with 93.3 to 94.1% [37], and in 2009 from healthy broilers with 92.8 to 93.6% nt identity [12].

Additional query cover and available sequences are needed to understand mutation rates, temporal, geographical and hosts patterns for RVA and non-RVA species of avian RV for the VP6 gene [35, 38]. Although VP6 is the most numerous and conserved protein of RV, sequence data analysis can elucidate and provide epidemiological aspects and knowledge regarding avian RV infections in the several avian hosts and the interaction with commercial poultry broilers from the highest production areas.

RV is an enteric infection that affects chicks in the first weeks of their lives, causing diarrhea and dehydration [5]. The previously mentioned factors adversely affect the batch, including reduced body weights, higher feed conversion rates, decreased uniformity, lower livability, and increased risk of secondary diseases [13]. Depending on the severity of the infection, economic losses can be substantial [10, 48].

Overall, the molecular characterization of the VP6 gene from avian RVA, RVD, and RVF strains contributes to a better understanding of the genetic diversity and epidemiology of RV infections in young broiler chickens in Brazil.

## Study limitations

This study has some limitations, including restricted geographic sampling, the exclusive analysis of young broiler chickens with clinical signs, and the use of partial VP6 gene sequences. Consequently, the results may not fully represent the genetic diversity of avian rotaviruses circulating among other hosts, age groups, or geographic regions.

## Data Availability

The datasets generated or analyzed during the study are available from the corresponding author upon reasonable request.
